# Pure discrete spectrum and regular model sets on some non-unimodular substitution tilings

**DOI:** 10.1107/S2053273322006714

**Published:** 2022-08-12

**Authors:** Jeong-Yup Lee

**Affiliations:** a Department of Mathematics Education, Catholic Kwandong University, Gangneung 25601, Republic of Korea; Universidad del País Vasco, Spain

**Keywords:** pure discrete spectrum, regular model sets, non-unimodular substitution, Pisot family substitution, Meyer sets

## Abstract

Substitution tilings with pure discrete spectrum are characterized as regular model sets whose cut-and-project scheme has an internal space that is a product of a Euclidean space and a profinite group. Assumptions made here are that the expansion map of the substitution is diagonalizable and its eigenvalues are all algebraically conjugate with the same multiplicity. A difference from the result of Lee *et al.* [*Acta Cryst.* (2020), A**76**, 600–610] is that unimodularity is no longer assumed in this paper.

## Abstract 2

The equivalence between pure discrete spectrum and regular model sets on some non-unimodular substitution tilings is established. This will help to provide useful information about the cut-and-project scheme used in the description of quasiperiodic structures.

## 1. Introduction

There has been considerable success in studying the structure of tilings with pure discrete spectrum by setting them in the context of model sets (Baake & Moody, 2004 ▸; Baake *et al.*, 2007 ▸; Strungaru, 2017 ▸; Akiyama *et al.*, 2015 ▸). However, in general settings, the relation between pure discrete spectrum and model sets is not completely understood and the cut-and-project scheme is usually constructed with an abstract internal space (Baake & Moody, 2004 ▸; Strungaru, 2017 ▸). Thus it is not easy to understand this relation concretely and get information about the structure from the relation. The notion of inter model sets was introduced by Baake *et al.* (2007 ▸) and Lee & Moody (2006 ▸) and we know the equivalence between pure discrete spectrum and inter model sets in substitution tilings (Lee, 2007 ▸). But there are still some limitations in getting useful information about the cut-and-project scheme (CPS) because the internal space was constructed abstractly. What is the internal space concretely? There was some progress in this direction by Lee *et al.* (2018 ▸) and Lee, Akiyama & Lee (2020 ▸). However, these papers make various assumptions about substitution tilings such as the expansion map is diagonalizable, the eigenvalues of the expansion map should be algebraically conjugate, the multiplicity of the eigenvalues should be the same, and the expansion map is unimodular. From a long perspective, we aim to gradually eliminate assumptions one by one. As a first step, in this paper we eliminate the assumption of unimodularity.

Our work was inspired by an example of Baake *et al.* (1998 ▸), which offers a guide to what the internal space should be. We will look at this in Example 5.10. The present paper is an extension of the result of Lee, Akiyama & Lee (2020 ▸) in the sense that the unimodularity condition is removed, and the setting is quite similar.

There are various research works on non-unimodular substitution cases (Baker *et al.*, 2006 ▸; Ei *et al.*, 2006 ▸; Siegel, 2002 ▸) that study symbolic substitution sequences or their geometric substitution tilings in dimension 1. Our definition of non-unimodularity looks slightly different from that defined in those papers. However, if we restrict the substitution tilings to one dimension 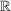 , the two definitions are the same.

We have four basic assumptions about a primitive substitution tiling 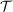 on 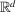 with an expansion map ϕ:

(i) ϕ is diagonalizable.

(ii) All the eigenvalues of ϕ are algebraically conjugate.

(iii) All the eigenvalues of ϕ have the same multiplicity.

(iv) 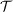 is rigid [see (14) for the definition].

We call these assumptions DAMR. This paper relies heavily on the rigid structure of substitution tilings, and the rigidity property is only known under those assumptions (i), (ii), (iii) together with finite local complexity (Theorem 2.9[Statement theorem2.9]). In Section 2, we review some definitions and known results that are going to be used in this paper. The main result of this paper shows the following:

Theorem 1.1 Let 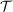 be a repetitive primitive substitution tiling on 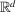 with a diagonalizable expansion map ϕ whose eigenvalues are algebraic conjugates with the same multiplicity and let 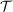 be rigid. If 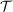 has pure discrete spectrum, then control point set 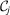 of each tile type is a regular model set in the CPS with an internal space which is a product of a Euclidean space and a profinite group, where 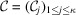 is a control point set of 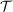 defined in (7) and the CPS is defined in (35).

In Section 3, we give an outline of the proof of this theorem in some simple case of substitution tilings with expansion map ϕ satisfying the DAMR assumptions defined above. In Section 4, we define an appropriate internal space and construct a CPS under the DAMR assumptions. Then we discuss the projected point sets 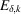 of neighbourhood bases of a topology in the internal space. In Section 6, under the assumption of pure discrete spectrum of 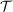 , we look at how the projected point sets 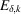 and the translation vector set Ξ of the same types of tiles in 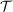 are related [see (8)]. Using the equivalent property ‘algebraic coincidence’ for pure discrete spectrum, we provide arguments to show that we actually have regular model sets.

## 2. Definitions and known results

We consider a primitive substitution tiling 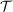 on 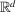 with expansion map ϕ satisfying the DAMR assumptions defined above. In this section, we recall some definitions and results that we are going to use in the later sections.

### 2.1. Tilings

We consider a set of types (or colours) 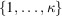 , which we fix once and for all. A *tile* in 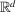 is defined as a pair 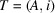 where 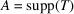 (the support of *T*) is a compact set in 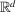 , which is the closure of its interior, and 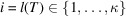 is the type of *T*. A *tiling* of 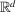 is a set 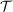 of tiles such that 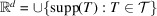 and distinct tiles have disjoint interiors.

Given a tiling 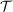 , a finite set of tiles of 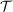 is called a 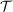 -*patch*. Recall that a tiling 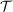 is said to be *repetitive* if the occurrence of every 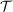 -patch is relatively dense in space. We say that a tiling 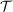 has *finite local complexity* (FLC) if for every 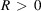 there are only finitely many translational classes of 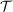 -patches whose support lies in some ball of radius *R* up to translations.

### 2.2. Delone κ-sets

A κ-*set* in 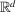 is a subset 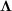 = 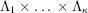 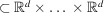 (κ copies) where 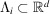 and κ is the number of colours. We also write 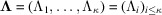 . Recall that a Delone set is a relatively dense and uniformly discrete subset of 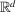 . We say that 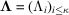 is a *Delone κ-set* in 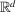 if each 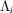 is Delone and 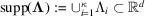 is Delone. The type (or colour) of a point *x* in the Delone κ-set 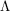 is *i* if 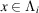 with 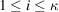 .

A Delone set Λ is called a *Meyer set* in 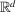 if 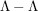 is uniformly discrete, which is equivalent to saying that 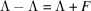 for some finite set *F* (see Meyer, 1972 ▸; Lagarias, 1996 ▸; Moody, 1997 ▸). If 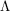 is a Delone κ-set and 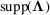 is a Meyer set, we say that 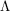 is a Meyer κ-set.

### 2.3. Substitutions

We say that a linear map 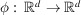 is *expansive* if there is a constant 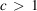 with 

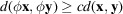

for all 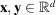 under some metric *d* on 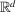 compatible with the standard topology.

Definition 2.1 Let 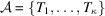 be a finite set of tiles on 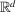 such that 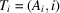 ; we will call them *prototiles*. Denote by 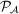 the set of patches made of tiles each of which is a translate of one of the 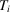 ’s. We say that 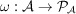 is a *tile-substitution* (or simply *substitution*) with an expansive map ϕ if there exist finite sets 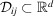 for 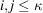 , such that 

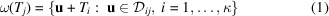

with 

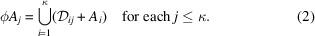

Here all sets in the right-hand side must have disjoint interiors; it is possible for some of the 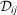 to be empty.

The substitution (1) is extended to all translates of prototiles by 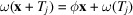 , and to patches and tilings by 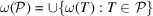 . The substitution ω can be iterated, producing larger and larger patches 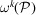 . A tiling 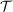 satisfying 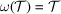 is called a *fixed point of the tile-substitution* or a *substitution tiling with expansion map* ϕ. It is known (and easy to see) (Solomyak, 1997 ▸) that one can always find a periodic point for ω in the tiling dynamical hull, *i.e.* 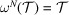 for some 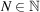 . In this case we use 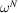 in the place of ω to obtain a fixed point tiling. The *substitution* 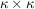 matrix 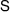 of the tile-substitution is defined by 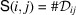 . We say that the substitution tiling 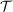 is *primitive* if there is an 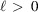 for which 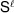 has no zero entries, where 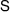 is the substitution matrix.

When there exists a monic polynomial 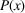 over 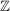 with the minimal degree satisfying 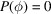 , we call the polynomial the *minimal polynomial of* ϕ *over* 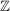 . We say that ϕ is *unimodular* if the minimal polynomial of ϕ over 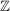 has constant term 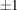 ; that is to say, the product of all roots of the minimal polynomial of ϕ is 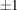 . If the constant term in the minimal polynomial of ϕ is not 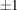 , then we say that ϕ is *non-unimodular*.

Note that for 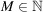 , 

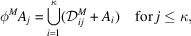

where 

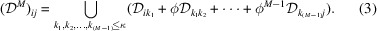

Definition 2.2

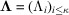
 is called a *substitution Delone κ-set* if 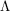 is a Delone κ-set and there exist an expansive map 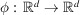 and finite sets 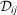 for 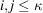 such that 

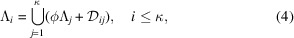

where the unions on the right-hand side are disjoint.

Definition 2.3 For a substitution Delone κ-set 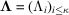 satisfying (4), define a matrix 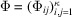 whose entries are finite (possibly empty) families of linear affine transformations on 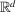 given by 

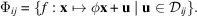

Define 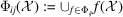 for 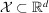 . For a κ-set 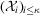 let 

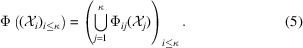

Thus 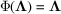 by definition. We say that Φ is a κ-*set substitution*. Let 

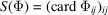

denote the *substitution matrix* corresponding to Φ.

Definition 2.4 (Mauduit, 1989 ▸.) An algebraic integer θ is a real Pisot number if it is greater than 1 and all its Galois conjugates are less than 1 in modulus, and a complex Pisot number if every Galois conjugate, except the complex conjugate 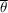 , has modulus less than 1. A set of algebraic integers 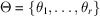 is a *Pisot family* if for every 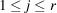 , every Galois conjugate η of 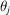 , with 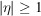 , is contained in Θ.

For 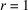 , with 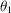 real and 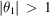 , this reduces to 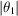 being a real Pisot number, and for 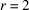 , with 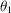 non-real and 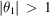 , to 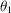 being a complex Pisot number.

### 2.4. Pure discrete spectrum and algebraic coincidence

Let 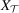 be the collection of tilings on 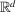 each of whose patches is a translate of a 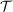 -patch. In the case that 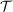 has FLC, there is a well known metric δ on the tilings: for a small 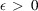 two tilings 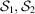 are ε-close if 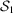 and 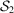 agree on the ball of radius 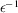 around the origin, after a translation of size less than ε (see Schlottmann, 2000 ▸; Radin & Wolff, 1992 ▸; Lee *et al.*, 2003 ▸). Then 

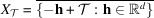

where the closure is taken in the topology induced by the metric δ.

It is known that a dynamical system 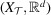 with a primitive substitution tiling 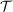 always has a unique ergodic measure μ in the dynamical system 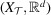 (see Solomyak, 1997 ▸; Lee *et al.*, 2003 ▸). We consider the associated group of unitary operators 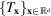 on 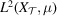 : 

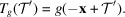

Every 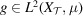 defines a function on 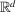 by 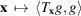 . This function is positive definite on 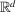 , so its Fourier transform is a positive measure 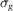 on 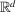 called the *spectral measure* corresponding to *g*. The dynamical system 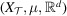 is said to have *pure discrete spectrum* if 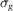 is pure point for every 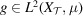 . We also say that 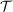 has pure discrete spectrum if the dynamical system 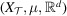 has pure discrete spectrum.

The notion of pure discrete spectrum of the dynamical system is quite closely connected wtih the notion of algebraic coincidence in Definition 2.6[Statement definition2.6]. For this we start by introducing control points. There is a standard way to choose distinguished points in the tiles of a primitive substitution tiling so that they form a ϕ-invariant Delone κ-set. They are called *control points* (Thurston, 1989 ▸; Praggastis, 1999 ▸), which are defined below.

Definition 2.5 Let 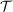 be a primitive substitution tiling with an expansion map ϕ. For every 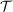 -tile *T*, we choose a tile 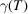 in the patch 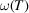 ; for all tiles of the same type in 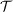 , we choose 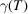 with the same relative position [*i.e.* if 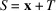 for some two tiles 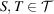 then 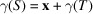 ]. This defines a map 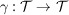 called the *tile map*. Then we define the *control point* for a tile 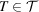 by 

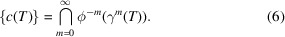

The control points satisfy the following: (*a*) 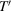 = 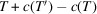 , for any tiles 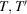 of the same type; (*b*) 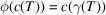 , for 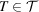 .

Let 

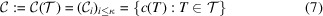

be a set of control points of the tiling 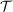 in 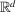 . Let us denote 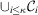 by 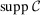 .

For tiles of any tiling 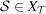 , the control points have the same relative position as in 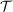 -tiles. The choice of control points is non-unique, but there are only finitely many possibilities, determined by the choice of the tile map. Let 

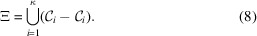

Since the substitution tiling 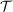 is primitive, it is possible to assume that the substitution matrix 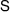 is positive taking 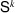 if necessary. So we consider a tile map 

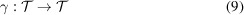

with the property that for every 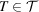 , the tile 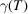 has the same tile type in 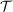 . That is to say, for every 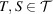 , 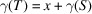 , where 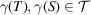 and 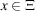 . Then for any 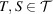 , 

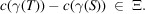




In order to have 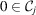 for some 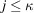 and 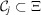 , we define the tile map as follows. It is known that there exists a finite generating patch 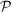 for which 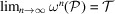 (Lagarias & Wang, 2003 ▸). Although it was defined there for primitive substitution *point sets*, it is easy to see that the same property holds for primitive substitution tilings. We call the finite patch 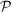 the *generating tile set*. When we apply the substitution infinitely many times to the generating tile set 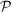 , we obtain the whole substitution tiling. So there exists 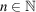 such that the *n*th iteration of the substitution to the generating tile set covers the origin. We choose a tile *R* in a patch 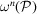 which contains the origin, where 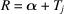 for some 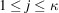 . Then there exists a fixed tile 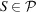 such that 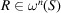 . Replacing the substitution ω by 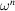 , we can define a tile map γ so that 

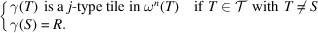

Then 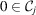 by the definition of the control point sets and so 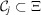 . Since 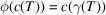 for any 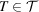 , 

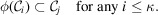

This implies that 

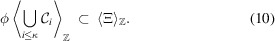

Definition 2.6 (Lee, 2007 ▸.) Let 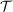 be a primitive substitution tiling on 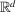 with an expansive map ϕ and let 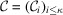 be a corresponding control point set. We say that 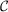 admits an *algebraic coincidence* if there exists 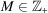 and 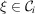 for some 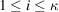 such that 

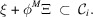

Note that if the algebraic coincidence is assumed, then for some 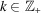 , 

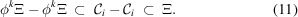

Theorem 2.7 [Theorem 3.13 (Lee, 2007 ▸), Theorem 2.6 (Lee, Akiyama & Lee, 2020 ▸).] Let 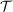 be a primitive substitution tiling on 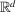 with an expansive map ϕ and 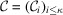 be a control point set of 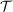 . Suppose that all the eigenvalues of ϕ are algebraic integers. Then 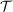 has pure discrete spectrum if and only if 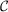 admits an algebraic coincidence.

### 2.5. CPS

We use a standard definition for a CPS and model sets (see Baake & Grimm, 2013 ▸). For convenience, we give the definition for our setting.

Definition 2.8 A *CPS* consists of a collection of spaces and mappings as follows: 

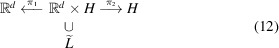

where 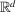 is a real Euclidean space, *H* is a locally compact Abelian group, 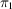 and 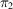 are the canonical projections, 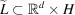 is a lattice, *i.e.* a discrete subgroup for which the quotient group 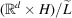 is compact, 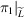 is injective, and 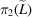 is dense in *H*. For a subset 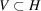 , we define 

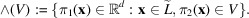

Here the set *V* is called *a window* of 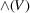 . A subset 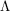 of 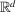 is called a *model set* if 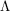 can be of the form 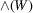 , where 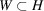 has non-empty interior and compact closure in the setting of the CPS in (12). The model set 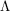 is *regular* if the boundary of *W*


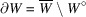

is of (Haar) measure 0. We say that 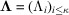 is a *model κ-set* (respectively, *regular model κ-set*) if each 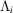 is a model set (respectively, regular model set) with respect to the same CPS.

### 2.6. Rigid structure on substitution tilings

The structure of a module generated by the control points is known only for the diagonalizable case for ϕ whose eigen­values are algebraically conjugate with the same multiplicity given by Lee & Solomyak (2012 ▸). We need to use the structure of the module in the subsequent sections. Thus we will have the same assumptions.

Let *J* be the multiplicity of each eigenvalue of ϕ and assume that the number of distinct eigenvalues of ϕ is *m*. For 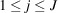 , we define 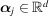 such that for each 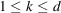 , 

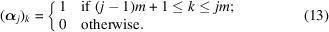

We recall the following theorem for the module structure of the control point sets. Although the theorem is not explicitly stated by Lee & Solomyak (2012 ▸), it can be read off from their Theorem 4.1 and Lemma 6.1.

Theorem 2.9 (Lee & Solomyak, 2012 ▸.) Let 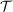 be a repetitive primitive substitution tiling on 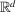 with an expansion map ϕ. Assume that 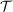 has FLC, ϕ is diagonalizable, and all the eigenvalues of ϕ are algebraically conjugate with the same multiplicity *J*. Then there exists a linear isomorphism 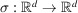 such that 

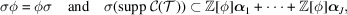

where 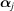 , 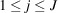 , are given as (13) and 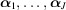 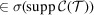 .

Note here that 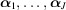 are linearly independent over 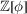 . A tiling 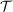 is said to be *rigid* if 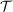 satisfies the result of Theorem 2.9[Statement theorem2.9]; that is to say, there exists a linear isomorphism 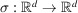 such that 

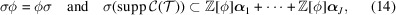

where 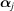 , 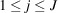 , are given as (13).

**Figure 1:**
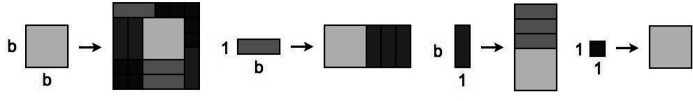
The Frank–Robinson tiling substitution.

As an example of a substitution tiling with the rigidity property, let us look at the Frank–Robinson substitution tiling (Frank & Robinson, 2006 ▸) (Fig. 1 ▸).

Take the tile-substutition 

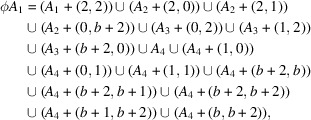




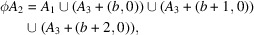




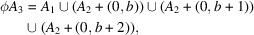




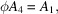

where *b* is the largest root of 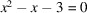 and

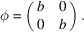

Then it gives a primitive substitution tiling 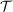 . Note that *b* is not a Pisot number. It was shown by Frank & Robinson (2006 ▸) that 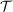 does not have FLC. One can observe that each set of translation vectors satisfies 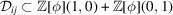 . Thus 

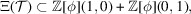

whence the rigidity holds.

## 3. Outline of the proof of Theorem 1.1[Statement theorem1.1]

We provide a brief outline of the proof of Theorem 1.1[Statement theorem1.1] for the simpler case of repetitive primitive substitution tilings 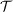 on 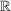 with an expansion factor λ ( 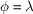 ):

(*a*) λ is non-unimodular,

(*b*) λ is a real Pisot number which is not an integer,

(*c*) 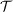 has FLC,

(*d*) 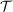 has pure discrete spectrum.

Let 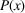 be the minimal polynomial of ϕ over 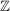 for which 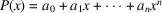 . Let 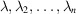 be all the roots of the equation 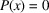 , where the absolute values of 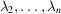 are all less than 1. Using the rigidity of Theorem 2.9[Statement theorem2.9], we get up to an isomorphism 

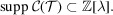

Using the algebraic conjugates 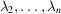 of λ whose absolute values are less than 1, we consider a Euclidean space 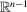 and the map 

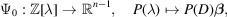

where 

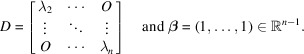

For the case of non-unimodular λ, we construct a profinite group below. We remark that if λ is unimodular, then the profinite group is trivial so that Theorem 1.1[Statement theorem1.1] can be covered by the work of Lee, Akiyama & Lee (2020 ▸). Let 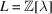 . From the non-unimodularity of λ, 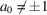 . So 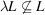 . Note that 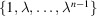 is a basis of *L* as a free 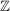 -module. Consider the map 

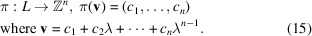

This gives an isomorphism of the 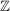 -module between *L* and 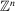 . Let 

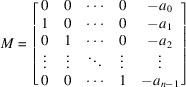

be the companion matrix of 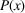 . Then 

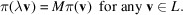

Notice that *M* acts on 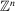 and the roots of the minimal polynomial of *M* over 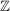 are exactly 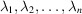 . Since 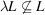 , 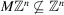 . Here we consider a profinite group 

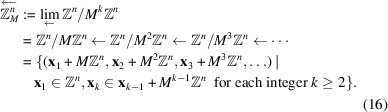

Since 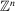 embeds in 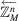 , we can identify 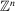 with its image in 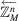 . Consider the following map:

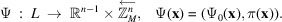

Now we construct a CPS whose physical space is 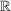 and internal space is 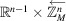 :[Chem scheme2] 
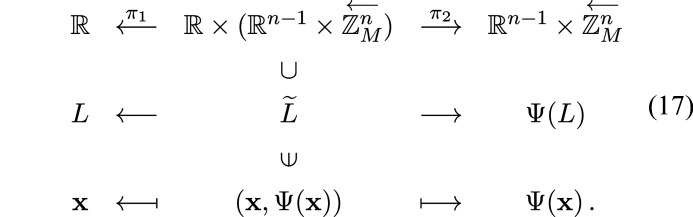


Under the assumption of pure discrete spectrum of 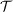 , we know that an algebraic coincidence occurs by Theorem 2.7[Statement theorem2.7]. So there exist 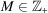 and 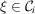 for some 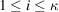 such that 

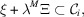

where Ξ is the set of translational vectors which translate a tile to the same type of tile in 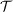 as given in (8). Notice that 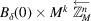 is a basis element in the locally compact abelian group 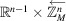 where 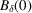 is a ball of radius δ around 0 in 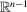 . We let 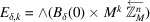 be the projected point set in 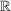 coming from a window 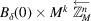 . It is important to understand the relation between 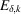 and Ξ. We discuss this in Section 4.2 (see also Lee *et al.*, 2018 ▸; Lee, Akiyama & Lee, 2020 ▸). From this relation, together with algebraic coincidence, we can view the control point set of 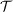 as a model set. Using Keesling’s argument (Keesling, 1999 ▸), we show that the control point set of 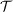 is actually a regular model set.

## 4. Construction of a CPS

We aim to prove that the structure of pure discrete spectrum in a substitution tiling can be described by a regular model set which comes from a CPS with the internal space that is a product of a Euclidean space and a profinite group. From Lee & Solomyak (2019 ▸), under the assumption of pure discrete spectrum, the control point set of the substitution tiling has the Meyer property and so has FLC. In general settings which are not substitution tilings, it is hard to expect that pure discrete spectrum implies neither the Meyer property nor FLC (Lee, Lenz *et al.*, 2020 ▸).

The setting that we consider here is a primitive substitution tiling 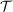 on 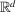 with an expansion map ϕ which satisfies the DAMR assumptions. Changing the tile substitution if necessary, we can assume that ϕ is a diagonal matrix without loss of generality.

Under the assumption of DAMR, it is also known from Lee & Solomyak (2012 ▸, 2019 ▸) that the control point set of the substitution tiling has the Meyer property if and only if the eigenvalues of ϕ form a Pisot family. In our setting, there is no algebraic conjugate η with 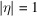 for the eigenvalues of ϕ, since ϕ is an expansion map. It is known that if ϕ is an expansion map of a primitive substitution tiling with FLC, every eigenvalue of ϕ is an algebraic integer (Kenyon & Solomyak, 2010 ▸; Kwapisz, 2016 ▸). Even for non-FLC cases, we know from the rigidity that the control point set lies in a finitely generated free abelian group *L* which spans 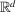 and 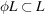 . So all the eigenvalues of ϕ are algebraic integers [Lemma 4.1 of Lee & Solomyak (2008 ▸)].

In the case of non-unimodular substitution tilings, there are two parts of spaces for the internal space of a CPS. One is a Euclidean part and the other is a profinite group part. We describe them below.

### 4.1. An internal space for a CPS

#### 4.1.1. Euclidean part for the internal space

In this subsection, we assume that there exists at least one algebraic conjugate whose absolute value is less than 1, which is different from the eigenvalues of ϕ. In the case of unimodular ϕ, we can observe that there always exists such an algebraic conjugate. But in the case of non-unimodular ϕ, it is possible not to have an algebraic conjugate whose absolute value is less than 1. For example, let us consider an expansion map 

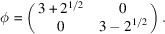

Then the minimal polynomial of ϕ is 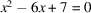 , which means that ϕ is non-unimodular. If there exists no other algebraic conjugate of the eigenvalues of ϕ whose absolute value is less than 1, one can skip this subsection and go to the next Section 4.1.2.

Recall that *J* is the multiplicity of the eigenvalues of ϕ, *d* is the dimension of the space 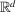 , *m* is the number of distinct eigenvalues of ϕ and 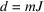 . We can write 

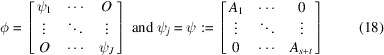

where 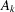 is a real 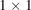 matrix for 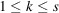 , a real 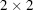 matrix of the form

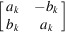

for 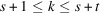 with 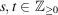 and 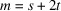 . Here 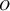 is the 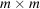 zero matrix and 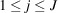 . Then the eigenvalues of ψ are 

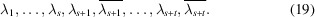

Note that *m* is the degree of the characteristic polynomial of ψ.

We assume that the minimal polynomial of ψ over 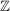 has *e* real roots and *f* pairs of complex conjugate roots. Since the minimal polynomial of ψ has the characteristic polynomial of ψ as a divisor, we can consider the roots of the minimal polynomial of ψ over 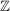 in the following order: 

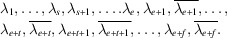

Let 

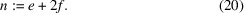

We now consider a Euclidean space whose dimension is 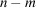 , whose number corresponds to the number of the other roots of the minimal polynomial of ψ which are not the eigenvalues of ψ. Let 

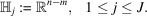

For 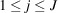 , define a 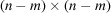 matrix 

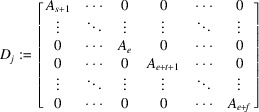

where 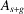 is a real 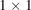 matrix with the value 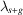 for 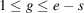 , and 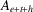 is a real 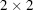 matrix of the form

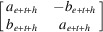

for 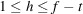 [see Lee, Akiyama & Lee (2020 ▸) for more details]. The matrix 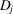 operates on the space 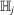 .

Notice that ϕ and ψ have the same minimal polynomial over 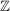 , since ϕ is the diagonal matrix containing *J* copies of ψ.

Let us consider now the following embeddings: 

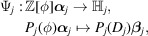

where 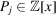 , 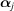 is as in (13), 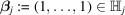 and 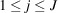 . Note that 

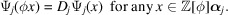

Let 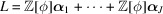 . Note that the minimal polynomial of ϕ is monic, since the eigenvalues of ϕ are all algebraic integers. So 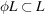 and 

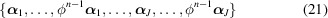

is a basis of *L* as a free 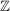 -module.

Now, we can define the map 

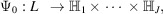




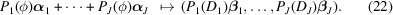

Since 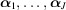 are linearly independent over 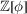 , the map 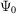 is well defined. Thus 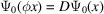 where 

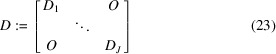

is a block diagonal 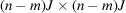 matrix in which 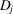 is an 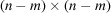 matrix, 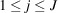 , and 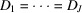 . Let 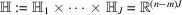 .

#### 4.1.2. Profinite group part for the internal space

To make the notation short, denote the basis of *L* given in (21) by 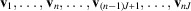 . Consider a 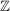 -module iso­morphism between *L* and 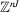




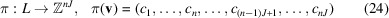

where 

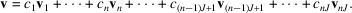

Consider the 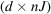 matrix:

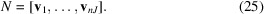

Since *L* spans 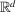 over 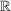 , the rank of *N* is *d*. Thus 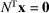 has only the trivial solution, where 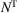 is the transpose of *N*. From 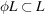 , we can write, for each 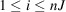 , 

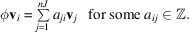

Let 

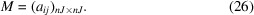

Notice that in a special case of 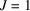 , *i.e.* 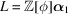 , *M* is the companion matrix of the minimal polynomial of ϕ over 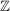 . Then 

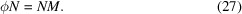

Note that for any 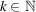 , 

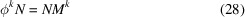

and

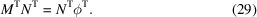

Notice also that for any 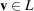 and for any 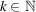 , 

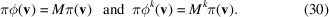

Lemma 4.1 Any eigenvalue of ϕ with multiplicity *J* becomes also the eigenvalue of *M* with the same multiplicity *J*. Furthermore the minimal polynomial of ϕ over 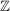 is the same as the minimal polynomial of *M* over 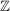 .

Proof Let λ be an eigenvalue of ϕ with multiplicity *J*. Since 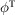 and ϕ have the same eigenvalues, λ is an eigenvalue of 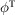 . Let 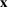 be the corresponding eigenvector of 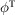 . Then 

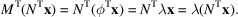

Since 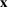 is nonzero, 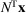 is nonzero and so λ is an eigenvalue of 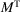 . Thus the eigenvalue λ of ϕ becomes also an eigenvalue of *M*. Since ϕ is a diagonal matrix, there are 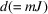 independent eigenvectors. The images of these vectors under 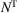 are the eigenvectors of 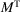 and linearly independent. Since all the eigenvalues of ϕ are algebraically conjugate with the same multiplicity *J*, all the eigenvalues of ϕ are also eigenvalues of 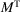 with the same multiplicity *J*. Thus we note that the set of the eigenvalues of *M* consists of all the eigenvalues of ϕ and all the other algebraic conjugates of them which are not the eigenvalues of ϕ, and the multiplicity of all the eigenvalues of *M* is *J*.

Since ϕ is a diagonal matrix and all the eigenvalues of ϕ are algebraic integers, there exists a minimal polynomial of ϕ over 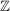 . Since *M* is an integer matrix, there exists a minimal polynomial of *M* over 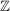 as well. Let 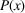 be the minimal polynomial of ϕ over 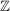 so that 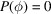 where 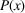 = 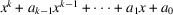 , 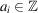 , and 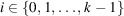 . Then using (30), for any 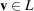 , 

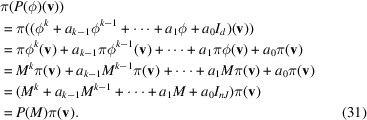

From (31), 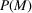 is a zero matrix. On the other hand, we can observe that if 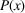 is the minimal polynomial of *M* over 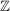 , then 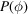 is a zero matrix as well. Thus the minimal polynomial of ϕ over 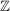 is the same as the minimal polynomial of *M* over 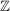 .

□

We can observe this property of Lemma 4.1[Statement lemma4.1] concretely with Example 5.10.

Let us consider the case that ϕ is non-unimodular, *i.e.* 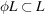 but 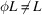 . Let us denote 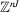 by 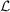 which is a lattice in 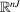 . Then 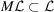 but 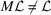 . We define the *M*-adic space which is an inverse limit space of 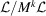 with 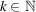 . Note that 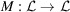 is an injective homomorphism. Observe that 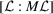 is non-trivial and finite. We have an inverse limit of an inverse system of discrete finite groups, 

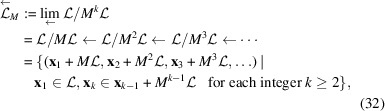

which is a profinite group. Note that 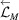 can be supplied with the usual topology of a profinite group. Note that for any element 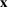 = 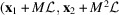 , 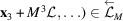 , 

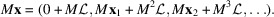

Thus it becomes a compact group which is invariant under the action of *M*. In particular, the cosets 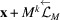 , 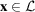 , 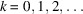 form a basis of open sets in 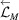 and each of these cosets is both open and closed. An important observation is that any two cosets in 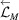 are either disjoint or one is contained in the other.

We let ρ denote the Haar measure on 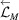 , normalized so that 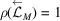 . Thus for a coset 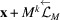 , 

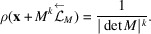

We define the translation-invariant metric *d* on 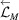 via 

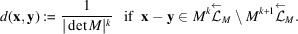

Note that 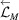 contains a canonical copy of 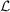 via the mapping 

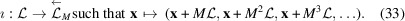

We can observe that 

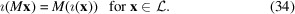

Note that 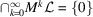 . So we can conclude that the mapping 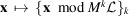 embeds 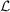 in 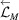 . We identify 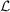 with its image in 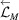 . Note that 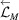 is the closure of 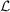 with respect to the topology induced by the metric *d*.

In the unimodularity case of ϕ, 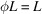 and so 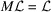 . Thus 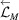 is trivial.

### 4.2. Concrete construction of a CPS

We construct a CPS taking 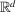 as a physical space and 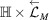 as an internal space. We will consider this construction dividing ϕ into three cases as given in the following remark. The following construction of a CPS has already appeared in the work of Minervino & Thuswaldner (2014 ▸) in the case of 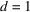 . Here we construct a CPS for the case of 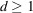 .

Remark 4.2 For an expansion map ϕ, there are three cases.

(i) If ϕ is unimodular, there exists at least one algebraic conjugate λ other than the eigenvalues of ϕ for which 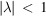 . Then the map ι in (33) is a trivial map and the internal space is constructed mainly by the Euclidean space discussed in Section 4.1.1.

(ii) If ϕ is non-unimodular and there exists no other algebraic conjugate of the eigenvalues of ϕ whose absolute value is less than 1, then 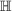 is a trivial group and the internal space is constructed exclusively by the profinite group (32) defined in Section 4.1.2.

(iii) If ϕ is non-unimodular and there exist algebraic conjugates (λ’s) other than the eigenvalues of ϕ for which 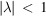 , then the internal space is a product of the Euclidean space in Section 4.1.1 and the profinite group in Section 4.1.2.

Let us define 

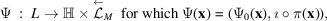

where π is defined as in (24). Let us construct a CPS:[Chem scheme3] 
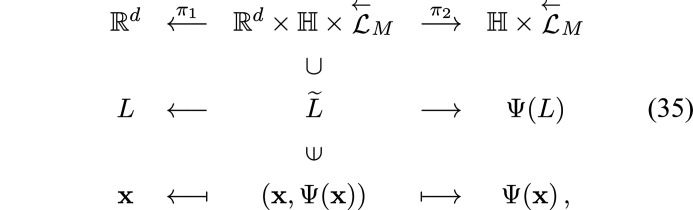


where 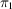 and 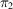 are canonical projections, 

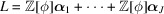

and 

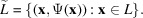

It is easy to see that 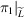 is injective. We shall show that 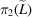 is dense in 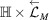 and 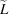 is a lattice in 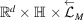 in Lemmas 4.3[Statement lemma4.3] and 4.4[Statement lemma4.4]. We note that 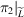 is injective, since Ψ is injective. Since ϕ commutes with the isomorphism σ in Theorem 2.9[Statement theorem2.9], we may identify the control point set 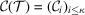 with its isomorphic image. Thus from Theorem 2.9[Statement theorem2.9], 

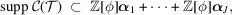

where 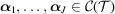 and 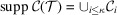 . Note that for any 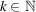 and 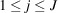 , 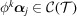 by the definition of the tile-map. So we can note that 

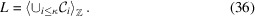

Lemma 4.3

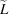
 is a lattice in 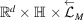 .

Proof For the case (i) of Remark 4.2[Statement enun4.2], 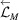 is trivial. So the statement of the lemma follows from Lemma 3.2 of Lee, Akiyama & Lee (2020 ▸).

For the case (ii) of Remark 4.2[Statement enun4.2], 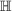 is trivial. Note that the matrix *M* in (26) is a 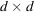 integer matrix and *L* is a lattice in 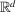 . So 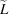 is a discrete subgroup of 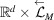 with respect to the product topology. Note that 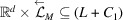 × 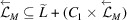 , where 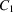 is a compact set in 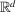 . Since 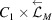 is compact, 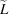 is relatively dense in 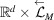 . Thus the statement of the lemma follows.

For the case (iii) of Remark 4.2[Statement enun4.2], let 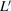 = 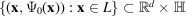 . In Lemma 3.2 of Lee, Akiyama & Lee (2020 ▸), we notice that the unimodularity property is used only in observing that 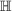 is not trivial in that paper. So by the same argument as Lemma 3.2 of Lee, Akiyama & Lee (2020 ▸), we obtain that 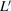 is a lattice in 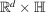 . This means that 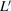 is a discrete subgroup such that 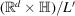 is compact. Notice that 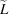 is still a discrete subgroup in 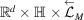 . Furthermore, 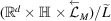 is compact. In fact, note that 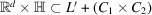 , where 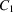 and 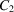 are compact sets in 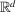 and 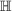 , respectively. Then 

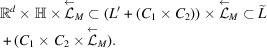

Since 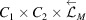 is compact, 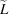 is relatively dense in 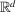 × 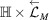 . Thus the statement of the lemma follows.

□

Lemma 4.4

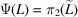
 and 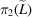 is dense in 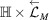 .

Proof For the case (i) of Remark 4.2[Statement enun4.2], 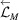 is trivial. So the statement of the lemma follows from Lemma 3.2 of Lee, Akiyama & Lee (2020 ▸).

For the case (ii) of Remark 4.2[Statement enun4.2], 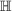 is trivial. Note that 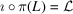 and 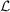 is dense in 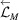 . Thus 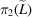 is dense in 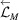 .

Let us consider the case (iii) of Remark 4.2[Statement enun4.2]. It is known from Lee, Akiyama & Lee (2020 ▸) that 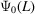 is dense in 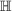 . For any open neighbourhood 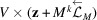 in 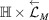 , there exists 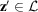 such that 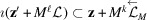 for some 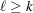 . Since 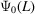 is dense in 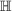 and 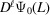 = 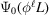 , 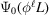 is dense in 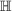 . Note that 

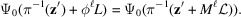

So 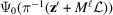 is dense in 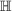 , where π is defined in (24). So 

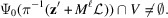

Hence 

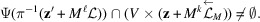

Thus 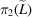 is dense in 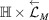 .

□

Now that we have proved that (35) is a CPS, we would like to introduce a special projected set 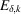 which will appear in the proof of the main result in Section 5. For 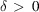 and 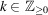 , we define 

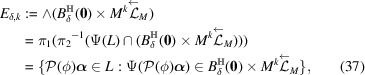

where 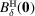 is an open ball around 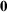 with a radius δ in 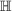 and 

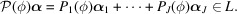

In the following lemma, we find an adequate window for a set 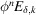 and note that 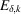 is a Meyer set.

Lemma 4.5 For any 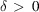 and 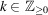 , let 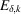 = 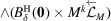 . Then for 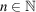 , 

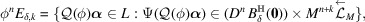

where 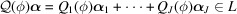 and 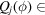 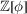 with 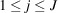 . Furthermore 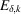 forms a Meyer set.

Proof Note that

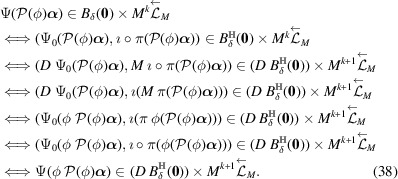

The third equivalence comes from (34) and the fourth equivalence comes from (30). Thus 

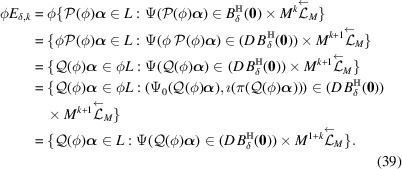

In the unimodularity case of ϕ, 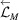 is trivial and 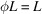 . So the last equality (39) follows. In the non-unimodularity case of ϕ, 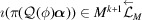 implies 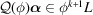 . Since 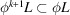 , 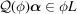 . This shows the last equality (39). Hence for any 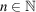 , 

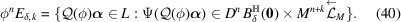

Since (35) is a CPS, 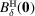 is bounded, and 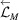 is compact, 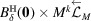 has a non-empty interior and compact closure, 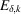 is a model set for each 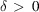 and 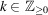 . It is given by Moody (1997 ▸) and Meyer (1972 ▸) that a model set is a Meyer set. Thus 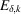 forms a Meyer set for each 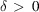 and 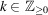 .

□

## 5. Main result

Recall that we consider a primitive substitution tiling 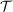 on 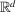 with a diagonal expansion map ϕ whose eigenvalues are algebraically conjugate with the same multiplicity *J* and 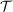 is rigid.

Under the assumption of the rigidity of 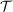 , the pure discrete spectrum of 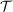 implies that the set of eigenvalues of ϕ forms a Pisot family [Lemma 5.1 (Lee & Solomyak, 2012 ▸)]. Recall that 

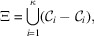

where 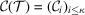 is a control point set of 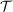 .

Lemma 5.1 Assume that ϕ satisfies the Pisot family condition. Then 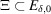 for some 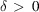 , where 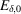 is given in (37).

Proof Notice that the setting for 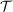 fulfils the conditions to use Lemma 4.5 of Lee & Solomyak (2008 ▸). So from this lemma, for any 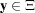 , 

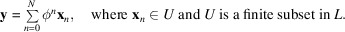

Recall that ϕ is an expansive map and satisfies the Pisot family condition. If there exists at least one algebraic conjugate λ other than the eigenvalues of ϕ for which 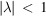 , 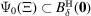 for some 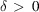 . So 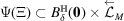 . If there exists no other algebraic conjugate of the eigenvalues of ϕ whose absolute value is less than 1, 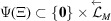 . From the definition of 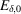 in (37), 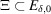 .

□

Lemma 5.2 Assume that 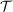 has pure discrete spectrum. Then for any 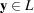 , there exists 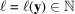 such that 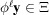 .

Proof Note from (36) that for any 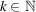 and 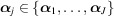 , 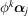 is contained in Ξ. Recall that 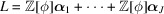 . From (10) and (36), 

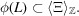

So for any 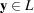 , 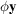 is a linear combination of 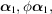 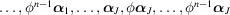 over 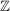 . Applying (11) many times if necessary, we get that for any 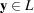 , 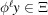 for some 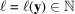 .

□

Proposition 5.3 Let 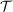 be a primitive substitution tiling on 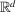 with an expansion map ϕ. Under the assumption of the existence of the CPS (35), if 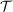 has pure discrete spectrum, then for any given 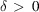 , there exists 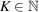 such that 

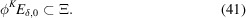

Proof Note that 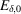 is a Meyer set and 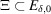 for some 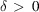 . Since Ξ is relatively dense, for any 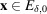 , there exists 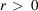 such that 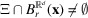 . It is important to note that from the Meyer property of 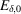 , the point set configurations 

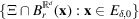

are finite up to translations. Let 

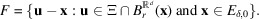

Then 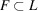 and *F* is a finite set. Thus for any 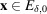 , 

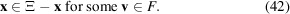

From Lemma 5.2[Statement lemma5.2], for any 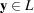 , there exists 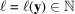 such that 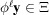 . Since 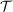 has pure discrete spectrum and so 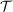 admits algebraic coincidence, by (11) there exists 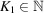 such that 

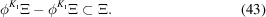

Applying the inclusion (43) finitely many times, we obtain that there exists 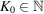 such that 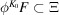 . Hence together with (42), there exists 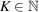 such that 

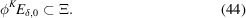

□

Proposition 5.4 Let 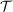 be a primitive substitution tiling on 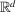 with a diagonalizable expansion map ϕ whose eigenvalues are algebraic conjugates with the same multiplicity and let 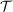 be rigid. Let Φ be the corresponding κ-set substitution of 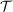 (see Definition 2.3[Statement definition2.3]). Suppose that 

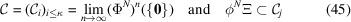

for some 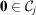 , 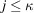 and 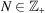 . Then each point set 

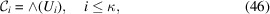

is a model set in the CPS (35) with a window 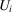 in 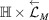 which is open and precompact.

Proof For each 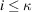 and 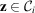 , there exist 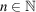 and 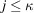 for which 

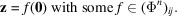

From 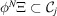 , 

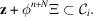

By Theorem 2.7[Statement theorem2.7] and Proposition 5.3[Statement proposition5.3], there exists 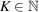 such that 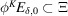 . Thus 

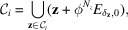

where 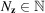 and 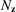 depends on 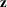 . Let 

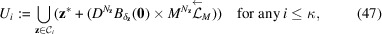

where 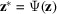 . Then for any 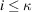




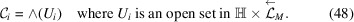

In (47), we assume that we have taken the minimal number 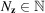 so that 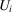 defined by using 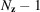 does not satisfy (48).

From Lemma 5.1[Statement lemma5.1], 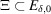 for some 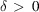 . Thus 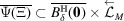 . Since 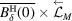 is compact, 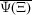 is compact. Thus 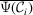 is compact.

□

Recall from Lagarias & Wang (2003 ▸) and Lee *et al.* (2003 ▸) that there exists a finite generating set 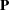 such that 

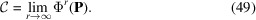

Since 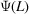 is dense in 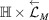 by Lemma 4.4[Statement lemma4.4], we have a unique extension of Φ to a κ-set substitution on 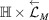 in the following way; if 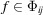 for which 

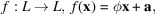

we define 

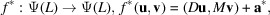

where 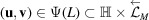 , *D* and *M* are given in (23) and (26), and 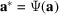 . If there is no confusion, we will use the same notation 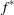 for the extended map.

Note that, by the Pisot family condition on ϕ, if there exists at least one algebraic conjugate λ other than the eigenvalues of ϕ for which 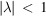 , there exists some 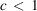 such that 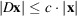 for any 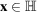 . Furthermore, from (33) 

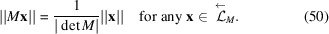

By the same argument as in Section 3 of Lee & Moody (2001 ▸), the κ-set substitution Φ induces a multi-component iterated function system on 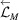 . Thus the κ-set substitution Φ determines a multi-component iterated function system 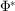 on 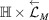 and 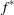 is a contraction on 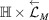 . Let 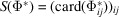 be a *substitution matrix* corresponding to 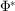 . Defining the compact subsets 

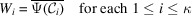

and using (5) and the continuity of the mappings, we have 

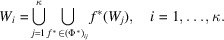

This shows that 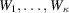 are the unique attractor of 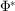 .

Lemma 5.5 Let 

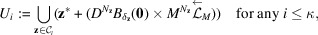

where 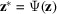 , as obtained in (47) with the minimal number 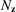 satisfying (48). For any 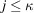 and any 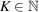 , we have 

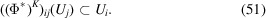

Proof For any 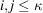 , 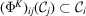 . Recall that 

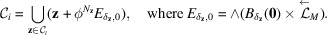

So for any 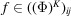 , 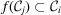 . Thus for any 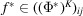 , 

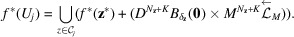

Notice that 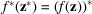 , where 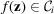 and 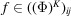 . Since we have taken the minimal number 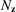 in (47) for each 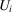 , 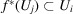 . Thus 

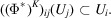

□

The following proposition shows that the Haar measure of 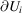 is zero for each 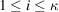 . This is proved using Keesling’s argument (Keesling, 1999 ▸).

Proposition 5.6 Let 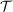 be a primitive substitution tiling on 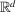 with a diagonalizable expansion map ϕ whose eigenvalues are algebraic conjugates with the same multiplicity and let 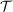 be rigid. Let Φ be the corresponding κ-set substitution of 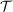 (see Definition 2.3[Statement definition2.3]). If 

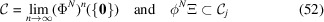

where 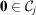 , 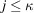 and 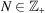 , then each model set 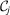 , 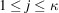 , has a window with boundary measure zero in the internal space 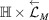 of CPS (35).

Proof Let us define 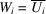 , where 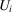 is the maximal open set in 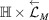 satisfying (46). From the assumption of (52), we first note that ϕ fulfils the Pisot family condition from Theorem 2.7[Statement theorem2.7] and Lemma 5.1 of Lee & Solomyak (2012 ▸). For every measurable set 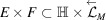 and for any 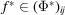 with 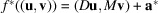 , 

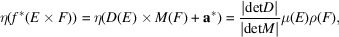

where μ is a Haar meaure in 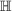 , ρ is a Haar measure in 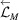 , 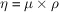 . Note that 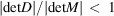 . In particular, 

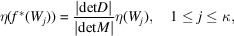

where 

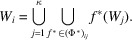

Let us denote 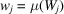 for 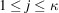 and 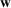 = 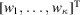 . Then for any 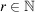 , 

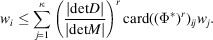

From Proposition 5.3[Statement proposition5.3], we know that 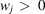 for any 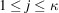 . Thus 

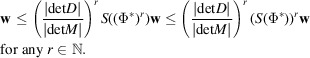

Note that the Perron–Frobenius eigenvalue of 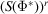 is 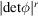 from Lagarias & Wang (2003 ▸). Since the minimal polynomials of ϕ and *M* over 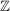 are the same from (27) and the multiplicities of eigenvalues of ϕ and *M* are the same from Lemma 4.1[Statement lemma4.1], we have 

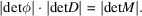

Since 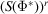 is a non-negative primitive matrix with Perron–Frobenius eigenvalue 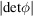 , from Lemma 1 of Lee & Moody (2001 ▸) 

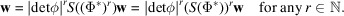

By the positivity of 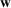 and 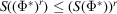 , 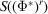 = 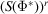 .

Recall that for any 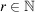 , 

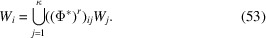

From (3), for any 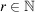 , 

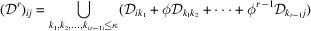

and 

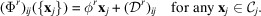

Note that 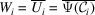 and 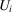 is a non-empty open set. As 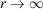 , 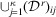 is dense in 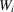 . We can find a non-empty open set 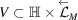 such that 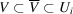 . So there exists 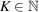 such that 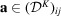 and 

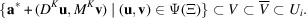

Since 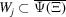 , 

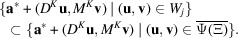

Thus there exists 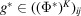 such that 

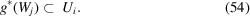

Hence 

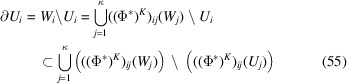




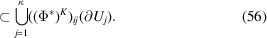

The inclusion (55) follows from Lemma 5.5[Statement lemma5.5]. Let 

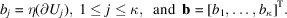

Then 

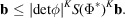

Thus from (54), there exists a matrix 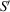 for which 

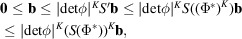

where 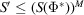 and 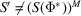 . If 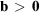 , again from Lemma 1 of Lee & Moody (2001 ▸), 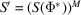 . This is a contradiction to (54). Therefore 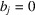 for any 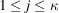 .

□

The regularity property of model sets is shared for all the elements in 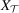 (see Schlottmann, 1998 ▸; Baake *et al.*, 2007 ▸; Lee & Moody, 2006 ▸). We state it in the following proposition.

Proposition 5.7 [(Schlottmann, 1998 ▸), Proposition 7 (Baake *et al.*, 2007 ▸), Proposition 4.4 (Lee & Moody, 2006 ▸).] Let 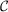 be a Delone κ-set in 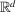 for which 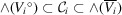 where 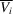 is compact and 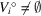 for 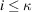 with respect to to some CPS. Then for any 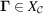 , there exists 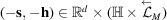 so that 

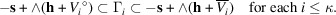

From the assumption of pure discrete spectrum and Remark 5.5 of Lee, Akiyama & Lee (2020 ▸), we can observe that the condition (52) is fulfilled in the following theorem.

Theorem 5.8 Let 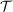 be a repetitive primitive substitution tiling on 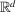 with a diagonalizable expansion map ϕ whose eigenvalues are algebraic conjugates with the same multiplicity and let 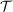 be rigid. If 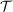 has pure discrete spectrum, then each control point set 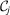 , 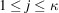 , is a regular model set in CPS (35) with an internal space which is a product of a Euclidean space and a profinite group.

Proof Through Section 4.1, we can construct the CPS (35) whose internal space is a product of a Euclidean space and a profinite group. Since 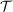 has pure discrete spectrum and is repetitive, we can find a substitution tiling 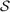 in 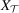 such that 

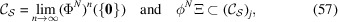

where 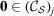 , 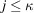 and 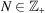 . From Propositions 5.3[Statement proposition5.3], 5.6[Statement proposition5.6] and 5.7[Statement proposition5.7], the statement of the theorem follows.

Corollary 5.9 Let 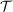 be a repetitive primitive substitution tiling on 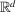 with a diagonalizable expansion map ϕ whose eigenvalues are algebraic conjugates with the same multiplicity and 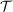 be rigid. Then 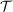 has pure discrete spectrum if and only if each control point set 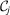 , 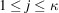 , is a regular model set in CPS (35) with an internal space which is a product of a Euclidean space and a profinite group.

Proof It is known that regular model sets have pure discrete spectrum in quite a general setting (Schlottmann, 2000 ▸). Together with Theorem 5.8[Statement theorem5.8], we obtain the equivalence between pure discrete spectrum and a regular model set in substitution tilings.

□

Now let us look at an example given by Baake *et al.* (1998 ▸).

*Example 5.10*. We look at the example of non-unimodular substitution tiling which is studied by Baake *et al.* (1998 ▸). This example is proven to be a regular model set in the setting of a CPS constructed by Baake *et al.* (1998 ▸). This has also been considered by Lee, Akiyama & Lee (2020 ▸), but it could only be described as a model set, not a regular model set. Here in the setting of CPS (35), we show that this example gives a regular model set. The substitution matrix of the primitive two-letter substitution 

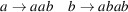

has the Perron–Frobenius eigenvalue 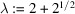 which is a Pisot number. A geometric substitution tiling arising from this substitution can be obtained by replacing symbols *a* and *b* in this sequence by the intervals of length 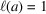 and 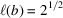 . Then we have the following tile-substitution ω 

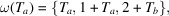




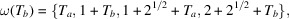

where 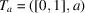 and 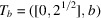 . Since 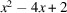 = 0 is the minimal polynomial of λ over 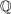 and the constant term of the polynomial is 2, the expansion factor λ is non-unimodular. Then we can construct a repetitive substitution tiling 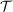 using the substitution ω.

From Theorem 2.9[Statement theorem2.9], we know that the control point set 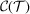 fulfils 

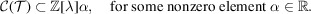

Let 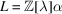 and 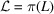 as in Section 4.1.2. Since 

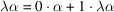




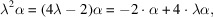

we get 

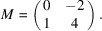

Recall from (32) 

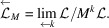

Let 

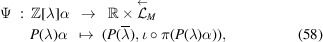

where 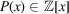 , 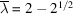 , and 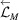 is a *M*-adic space. Since this substitution tiling is known to have pure discrete spectrum (see Baake *et al.*, 1998 ▸), it admits an algebraic coincidence. By Proposition 4.4 of Lee (2007 ▸) and rewriting the substitution, if necessary, we know that there exists a substitution tiling 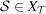 such that 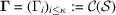 and 

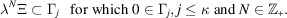

Then, by the same argument as in Proposition 5.4[Statement proposition5.4], 

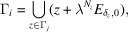

where 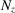 depends on *z* and 

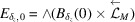

for some ball 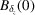 of radius 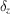 around 0 in 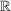 . Let 

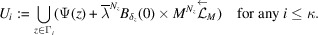

Thus 

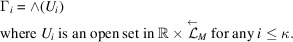

From Proposition 5.7[Statement proposition5.7], we can observe that the pure discrete spectrum of 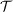 gives a model set with an open and precompact window in the internal space 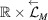 for the control point set 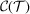 . From Proposition 5.6[Statement proposition5.6], the measures of the boundaries of the windows are all zero.

Now let us look at another example of a constant-length substitution tiling in 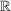 . This example shows that it is important to start with a control point set satisfying the containment (10).

*Example 5.11*. Consider a two-letter substitution defined as follows: 

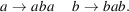

The expansion factor 3 and each prototile can be taken as a unit interval. Starting from 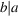 , we can expand *b* to the left-hand side and *a* to the right-hand side, applying the substitution infinite times. Then we get the following bi-infinite sequence:

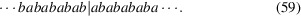

We consider two prototiles 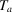 and 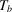 each of which corresponds to the letter *a* and the letter *b*. Following the sequence (59), we replace each letter by the corresponding prototile and obtain a substitution tiling 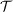 which is fixed under the substitution[Chem scheme1] 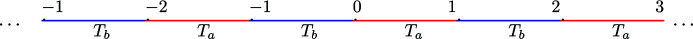


As a representative point of each tile, if one takes the left end of each interval in the tiling, one gets two point sets 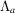 and 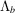 such that 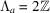 and 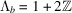 . Since 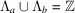 , we can take 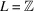 . Notice in this case that the Euclidean part for the internal space is trivial and the profinite group is 

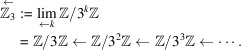

Notice that there does not exist 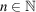 such that 

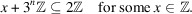

This means that neither 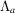 nor 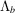 can be described as a model set projected from a window whose interior is non-empty in 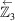 . However the substitution tiling 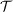 has pure discrete spectrum, since it is a periodic structure. The problem here is that the control point set 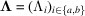 is not taken to satisfy the containment (10).

On the other hand, if we take the tile map 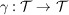 for which 

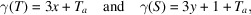

where 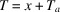 and 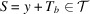 with 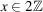 and 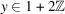 , then the control point set 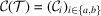 is 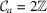 and 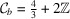 . So 

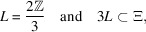

satisfying the containment (10), and the profinite group is 

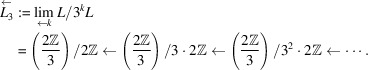

So

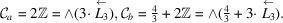

Therefore 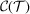 can be described as a model set.

## 6. Further study

In this paper, the rigid structure property of substitution tilings is used to make a connection from pure discrete spectrum to regular model sets, especially to compute the boundary measure of windows. So far, the rigid structure property is known for substitution tilings whose expansion maps (*Q*) are diagonalizable and the eigenvalues of *Q* are algebraically conjugate with the same multiplicity (Lee & Solomyak, 2012 ▸). Thus it would be useful to know some rigid structure for more general settings. If the rigidity property is precisely known for general substitution tilings, it is expected that we will be able to find the connection from pure discrete spectrum to regular model sets.
